# Improvements to the Gulf pipefish *Syngnathus scovelli* genome

**DOI:** 10.46471/gigabyte.76

**Published:** 2023-02-20

**Authors:** Balan Ramesh, Clay M. Small, Hope Healey, Bernadette Johnson, Elyse Barker, Mark Currey, Susan Bassham, Megean Myers, William A. Cresko, Adam Gregory Jones

**Affiliations:** ^1^ Department of Biological Sciences, University of Idaho, Moscow, ID 83844, USA; ^2^ Institute of Ecology and Evolution, University of Oregon, Eugene, OR 97403, USA; ^3^ Presidential Initiative in Data Science, University of Oregon, Eugene, OR 97403, USA

## Abstract

The Gulf pipefish *Syngnathus scovelli* has emerged as an important species for studying sexual selection, development, and physiology. Comparative evolutionary genomics research involving fishes from Syngnathidae depends on having a high-quality genome assembly and annotation. However, the first *S. scovelli* genome assembled using short-read sequences and a smaller RNA-sequence dataset has limited contiguity and a relatively poor annotation. Here, using PacBio long-read high-fidelity sequences and a proximity ligation library, we generate an improved assembly to obtain 22 chromosome-level scaffolds. Compared to the first assembly, the gaps in the improved assembly are smaller, the N75 is larger, and our genome is ~95% BUSCO complete. Using a large body of RNA-Seq reads from different tissue types and NCBI's Eukaryotic Annotation Pipeline, we discovered 28,162 genes, of which 8,061 are non-coding genes. Our new genome assembly and annotation are tagged as a RefSeq genome by NCBI and provide enhanced resources for research work involving *S. scovelli.﻿*

## Data description

This article presents a resource (genome assembly) that marks a technological improvement compared to the one previously published in the article, “The genome of the Gulf pipefish enables understanding of evolutionary innovations” [1].

A *de novo* genome assembly is evaluated based on three primary criteria: accuracy or correctness, completeness, and contiguity [2, 3]. Typically, the correctness of a genome is one of the most challenging features to measure. However, with modern, long-read sequencing technologies, the orientation of the contigs and the gene order of an assembly are highly accurate [4–6]. On the other hand, completeness and contiguity are easier to measure [6–8] yet more challenging to achieve, especially in non-model organisms. The Gulf pipefish (*Syngnathus scovelli*, NCBI:txid161590, fishbase ID: 3306) genome is an essential resource for the study of comparative genomics, evolutionary developmental biology, and other related topics [1, 9–15]. Given the technological constraints when it was initially sequenced, the first version of the *S. scovelli* genome is highly accurate and mostly complete, but it leaves considerable room for improvement with respect to contiguity [1]. Here, with the use of third-generation sequencing technology, including PacBio High Fidelity (Hi-Fi) long reads from circular consensus sequences (CCS) and Hi-C proximity ligation from Phase Genomics, we produced a nearly complete chromosome-scale genome assembly that not only improves completeness and accuracy but is also the most contiguous genome yet produced for the genus *Syngnathus* (Table 1).

**Table 1:** Contiguity metrics from QUAST for various *Syngnathus* species.

Metrics	*S. acus*	*S. rostellatus*	*S. typhle*	*S. floridae*	*S. scovelli* _v1	*S. scovelli* _v2
Number of contigs	87	8,935	526	6,895	886	526
Largest contig	28,444,102	856,273	9,665,359	61,807,209	23,505,159	30,098,933
Total length	324,331,233	280,208,023	313,958,489	303,298,972	305,995,683	431,750,762
Reference length	324,331,233	324,331,233	324,331,233	324,331,233	324,331,233	324,331,233
GC (%)	43.46	43.08	43.29	43.63	42.95	45.00
Reference GC (%)	43.46	43.46	43.46	43.46	43.46	43.46
N50	14,974,571	88,962	3,046,963	7,845,045	12,400,093	17,337,441
NG50	14,974,571	70,018	3,012,268	7,783,711	11,493,655	20,118,474
N75	11,896,884	34,357	1,098,273	21,150	8,458,319	13,347,818
NG75	11,896,884	15,229	998,421	17,023	7,908,134	15,901,424
L50	8	812	30	5	10	10
LG50	8	1,092	32	6	11	7
L75	14	2,068	72	1,160	17	17
LG75	14	3,492	79	2,003	19	12

For NGx and LGx calculations, *S. acus* was used as the reference species. All the *Sygnathus* genomes (except *S. scovelli*) were last accessed from NCBI on 2022-July-26.

### Context

Evolutionary novelties are widespread across the tree of life. However, the origin of *de novo* genes and their associated regulatory networks, as well as their effects on the phenotype, remain mysterious in most species. Syngnathidae is a family of teleost fishes that includes pipefishes, seahorses, and seadragons [1, 12–16]. Syngnathid fishes are known for their evolutionary novelty with respect to morphology and physiology. For instance, species in this family have variously evolved elaborate leafy appendages, male brooding structures, prehensile tails, elongated facial bones, and numerous other unusual traits [1, 12–14]. With a variety of mating systems and sex roles [12–16], the syngnathid fishes also provide an excellent study system to investigate the generality of theories on sexual selection and reproductive biology [15, 16]. Advances in comparative genomics and the evolutionary developmental biology of novel traits in syngnathids require the development of additional genomic tools. Among these are well-assembled and annotated genomes [1]. Here, we took a step in this direction by producing an improved reference genome for the Gulf pipefish.

## Methods

### DNA and RNA extraction

We collected *S. scovelli* from the Gulf of Mexico in Florida, USA (Tampa Bay), and flash froze them in liquid nitrogen. We pulverized approximately 50 mg of whole-body tissue (posterior to the urogenital opening) from a single male on liquid nitrogen, which we submitted to the University of Oregon Genomics and Cell Characterization Core Facility (UOGC3F) for high-molecular-weight DNA isolation using the PacBio Nanobind tissue kit. We submitted similar (but unpulverized) frozen tissue from the same individual fish to Phase Genomics to generate a Hi-C library using Proximo Animal (v4) technology.

In addition, we used organic extraction with TRIzol Reagent, followed by column-based binding and purification using the Qiagen RNeasy MinElute Cleanup Kit, to extract mRNA from the Brain, Eye, Gills, Muscle/Skin, Testis, Ovary, Broodpouch, and Flap tissues.

### Sequencing and assembly

After the size selection of genomic DNA using the Blue Pippin (11 kb cutoff), the UOGC3F constructed a sequencing library using the SMRTbell Express Template Prep Kit 2.0. One SMRT cell was sequenced by the UOGC3F using PacBio Sequel II technology, yielding 33.39 Gb in 2.05M CCS reads (out of 6.298M Hi-Fi reads in total). We sequenced 70.4 Gb of paired-end 150 nucleotide reads (234.6 million in total) from the Hi-C library using an Illumina NovaSeq 6000 at the UOGC3F. The RNA sequencing libraries were prepared using the KAPA mRNA HyperPrep Kit. We sequenced 159 bp paired-end reads using Illumina Novaseq 6000 for each tissue from the RNA sequencing libraries for annotation.

**Figure 1.**
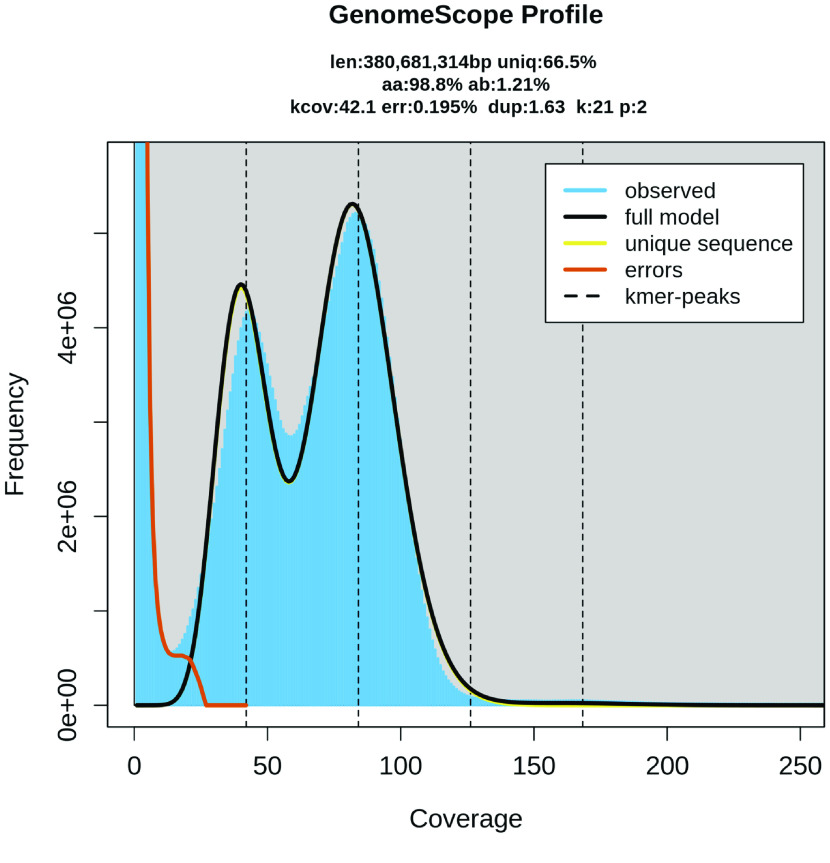
Estimated genome size of *Syngnathus scovelli* based on k-mer analysis using Meryl and Genomescope.

Using the Hi-Fi sequences, we estimated the genome size using genomescope2 (v2.0, RRID:SCR_017014) [17] and meryl (v2.2) [18] with a default k-mer size of 21 (Figure 1). The paired-end Hi-C reads were trimmed using trimmomatic (v0.39, RRID:SCR_011848) [19] with the parameter HEADCROP:1 to remove the first base, which was of low quality. Together with the Hi-Fi sequences, we assembled the first-pass genome assembly in Hi-C integrated mode using hifiasm (v0.16.1, RRID:SCR_021069) [18] with default parameters. The First-Pass assembly refers to the first draft consensus assembly from the Hi-Fi and Hi-C data. We extracted the consensus genome from hifiasm in fasta format and assembled the contigs into scaffolds using juicer (v1.6, RRID:SCR_017226) [20]. We used the 3D-DNA (version date: Dec 7, 2016) [21] pipeline to merely order the scaffolds. The Hi-C contact map of the ordered scaffolds was visualized using juicebox (v1.9.8, RRID:SCR_021172) with no breaking of the original contigs. 

### Assessment of completeness and contiguity

**Figure 2.**
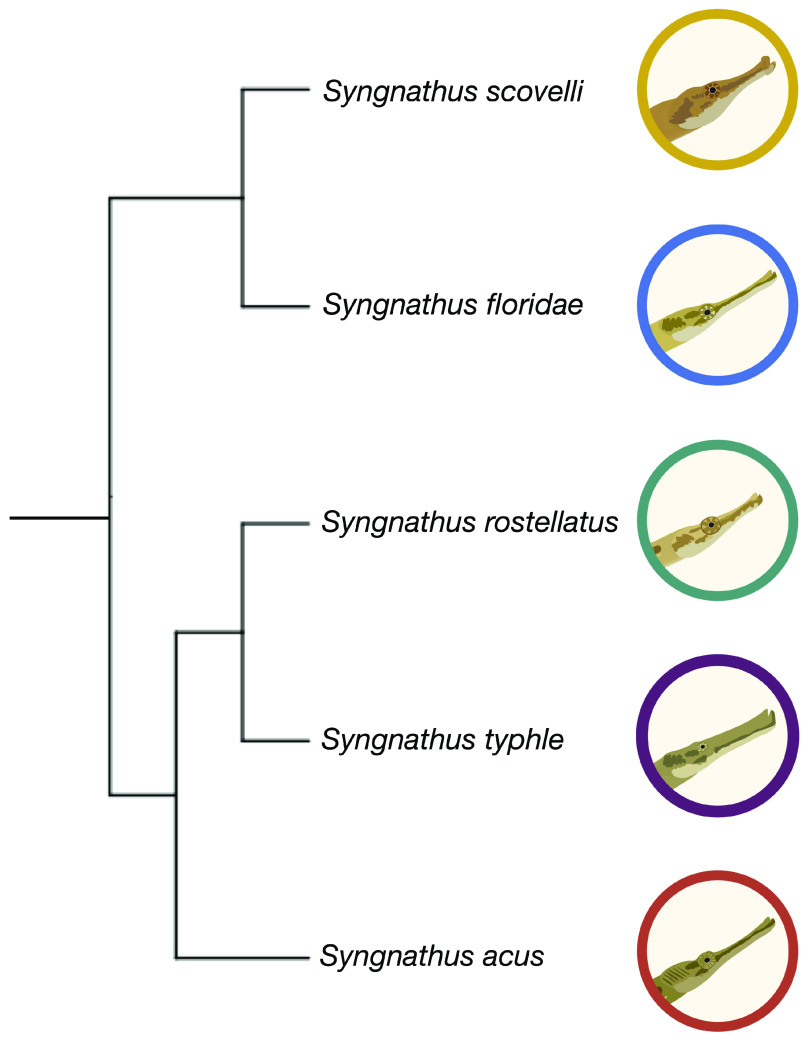
Cladogram of the five *Syngnathus* species in this study. This phylogeny is based on the Ultra Conserved Elements among all syngnathids [26].

To compare the completeness and contiguity of the latest version of the *S. scovelli* genome against the other *Syngnathus* genomes (Figure 2), we downloaded the genome assemblies of *S. acus* (GCA_024217435.2), *S. rostellatus* (GCA_901007895.1) [22], *S. typhle* (GCA_901007915.1) [22], and *S. floridae* (GCA_010014945.1) from NCBI. We used Benchmarking Universal Single-Copy Orthologs (BUSCO v5.2.2, RRID:SCR_015008) [23] in genome mode with the actinopterygii_odb10 database (as of 2021-02-19) to evaluate the completeness of the genome. Also, we used a k-mer-based assessment using Merqury (v2020-01-29, RRID:SCR_004231. [24]) to estimate the completeness and the base error rate. We then used the Quality Assessment Tool (QUAST v5.0.2, RRID:SCR_001228) [25] to estimate Nx and Lx statistics for our assembly.

### Annotation using the NCBI Eukaryotic annotation pipeline

The NCBI Eukaryotic Genome Annotation Pipeline (v10.0) is an automated software pipeline identifying coding and non-coding genes, transcripts, and proteins on complete and incomplete genome submissions to NCBI. The core components of this pipeline are the RNA alignment program (STAR and Splign) and Gnomon, a gene prediction program. In this pipeline, the RNA-Seq reads from the various (Brain, Eye, Gills, Muscle/Skin, Testis, Ovary, Broodpouch, and Flap) tissues of multiple samples, including the *S. scovelli* individual used for Hi-Fi and Hi-C sequence data (SRR20438584–SRR20438604), were aligned to the genome. Gnomon combines the information from alignments of the transcripts and the *ab initio* models from a Hidden Markov Model-based algorithm to create a RefSeq annotation. This RefSeq annotation produces a non-redundant set of a predicted transcriptome and a proteome that can be used for various analyses. The Eukaryotic annotation pipeline is not publicly available; thus, we requested the staff at NCBI to annotate the *S. scovelli* genome.

## Data validation and quality control

### Assembly statistics

**Figure 3.**
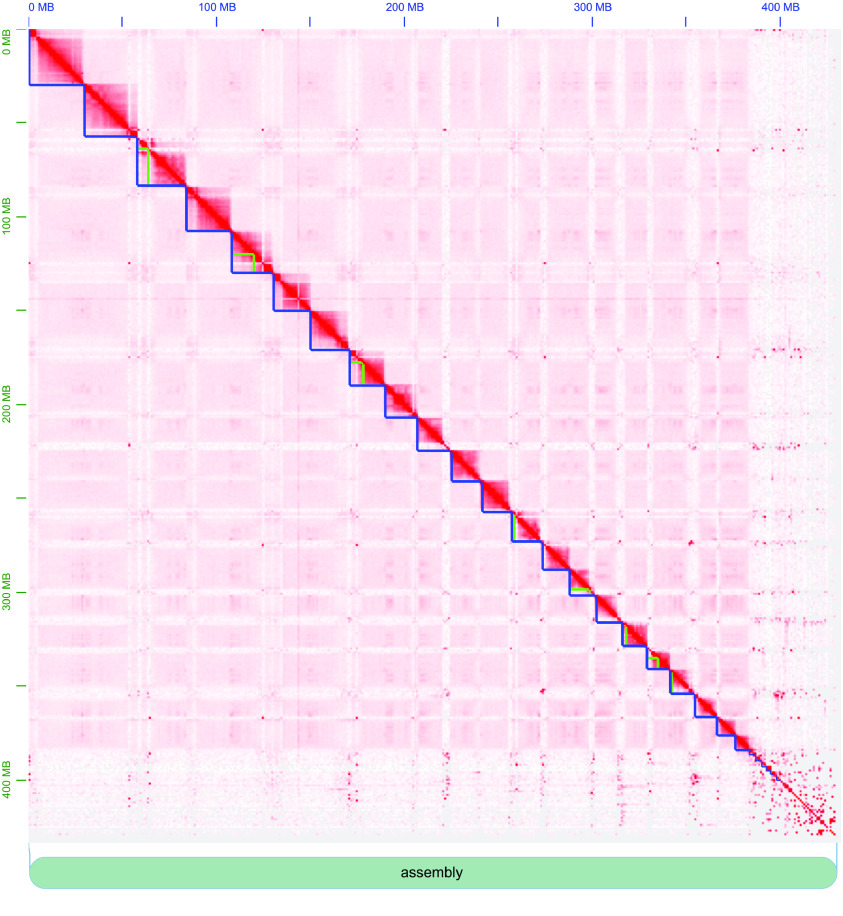
Visualization of contact maps from Hi-C reads for *Syngnathus scovelli* (v2). The first 22 primary assembly features (blue lines) sum to about 380 Mb in size, which is the estimated genome size for the species. The green lines reflect the individual contigs from the hifiasm assembly that were organized into chromosome-level scaffolds based on Hi-C contact data.

With approximately 2 million Hi-Fi reads and 234.6 million Hi-C reads, we generated the first pass consensus assembly with 585 contigs. The N50 and L50 for this assembly were 15.5 Mb and 11, respectively. We scaffolded this assembly to correct misassembles and produced a final assembly containing 526 contigs with N50 and L50 values of 17.3 Mb and 10, respectively (Table 1). This improved version of the *S. scovelli* genome has around three times fewer contigs compared to the original *S. scovelli* genome. The NG50 and NG75 are ∼1.75× and ∼2× larger, respectively, than the previous assembly, implying less fragmentation. Our new assembly reduces the number of gaps per 100 kilobase pairs (kb) from 6,837.20 Ns per 100 kb to a mere 0.27 Ns per 100 kbp, owing to the increased contiguity. This new *S. scovelli* genome is on par with the current best genome in the *Syngnathus* genus, that of *S. acus*, which is a complete chromosome-scale assembly. The first 22 scaffolds of the *S. scovelli* genome are of chromosome-scale in line with the genetic map [1] and the karyotype data [27] with a total length of around 380 Mb (Figure 3), comparable to the estimated genome size of 380 Mb (see GigaDB [28]; Table 2 and Figure 3). In addition, 88.94% of the total assembly length is captured in the 22 chromosome-scale scaffolds. For 15 of ﻿the chromosome-scale scaffolds, a single contig makes up the total length; the remaining seven are generally composed of a small number of contigs (Figure 3).

**Figure 4.**
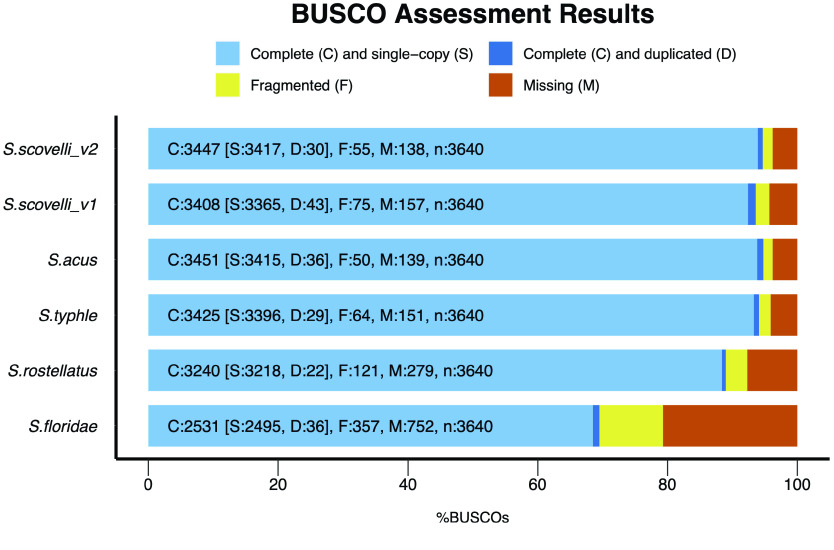
Comparison of BUSCO completeness among all the five *Syngnathus* species.

**Table 2:** Contiguity metrics from QUAST for the first pass and the scaffolded assembly of *S. scovelli* _v2.

Metrics	Haplotype1	Haplotype2	Primary consensus assembly	Scaffolded assembly
Number of contigs	901	544	585	526
Largest contig	21,671,036	23,661,123	30,098,933	30,098,933
Total length	427,545,154	428,155,884	431,749,582	431,750,762
GC (%)	44.99	44.78	45.00	45.00
N50	10,825,652	10,535,849	15,551,623	17,337,441
N75	4,999,310	4,477,557	11,049,644	13,347,818
L50	15	15	11	10
L75	29	30	19	17
Number of N’s per 100 kbp	0.00	0.00	0.00	0.27

### BUSCO and Merqury results

BUSCO results suggest a high degree of completeness as it found 95% of the orthologs in the Actinopterygii dataset (94.7*%* [*S*: 93.9*%*, *D*: 0.8*%*], *F*: 1.5*%*, *M*: 3.8*%*, *n*: 3,640) when run in genome mode (Figure 4) and the Merqury evaluation suggests that the genome is ∼86% complete with a ﻿quality value (QV) of 61.37 and an error rate of 7.3 × 10^−5^ *%* (see GigaDB [28] for more details; Tables 3 and 4).

**Table 3:** k-mer based assembly evaluation for completeness using Merqury.

Assembly	k-mer set used	solid k-mers in the assembly	Total solid k-mers in the read set	Completeness (%)
*S. scovelli* _v2	all	272,969,166	318,487,563	85.708

**Table 4:** k-mer based quality evaluation using Merqury.

Assembly	k-mers uniquely found only in the assembly	k-mers found in both the assembly and the read set	QV	Error rate
*S. scovelli*_v2	6,614	431,737,882	61.3697	7.29504 × 10^−7^

Consistent with the BUSCO contiguity metrics, the genome is on par with *S. acus* for completeness, which is also around 95% complete. Missing genes make up the majority of the remaining 5% of genes. We identified genes likely to be truly missing from the *S. scovelli* genome and more broadly from members of Syngnathidae (including the seahorses, genus *Hippocampus* along with *Syngnathus*) by confirming their absence across the BUSCO results from the present assembly, four additional members of the genus *Syngnathus*, and six additional *Hippocampus* publicly available assemblies (see GigaDB [28] for additional details). Of the missing BUSCO genes, 83 are shared among all the species of *Syngnathus*, and 38 are missing from both genera (see GigaDB [28] for additional details). Future work could profitably explore these missing genes, as some may be related to the interesting novel traits in syngnathid fishes.

### Annotation results

After masking about 43% of the genome, the annotations resulted in the prediction of about 28,162 genes, of which 8,061 are non-coding genes (see GigaDB [28]; Tables 5 and 6). The 28,162 genes produce about 59,938 transcripts, of which 47,846 are mRNA, and the rest is made up of other types of RNAs such as tRNA, lncRNA, and others. Out of the 20,101 coding genes, 18,616 had a protein with an alignment covering 50% or more of the query against the UniProtKB curated protein set, and 9,152 had an alignment covering 95% or more of the query.

**Table 5:** Gene and Feature Statistics from NCBI Eukaryotic Pipeline.

Feature	*S. scovelli*_v2
Genes and pseudogenes	29,062
protein-coding	20,101
non-coding	8,061
Transcribed pseudogenes	0
Non-transcribed pseudogenes	887
genes with variants	10,398
Immunoglobulin/T-cell receptor gene segments	9
other	4
mRNAs	47,846
fully-supported	47,491
with >5% *ab initio*	89
partial	39
with filled gap(s)	0
known RefSeq	0
model RefSeq	47,846
non-coding RNAs	12,092
fully-supported	7,318
with >5% *ab initio*	0
partial	5
with filled gap(s)	0
known RefSeq	0
model RefSeq	10,741
pseudo transcripts	0
fully-supported	0
with >5% *ab initio*	0
partial	0
with filled gap(s)	0
known RefSeq	0
model RefSeq	0
CDSs	47,855
fully-supported	47,491
with >5% *ab initio*	115
partial	39
with major correction(s)	144
known RefSeq	0
model RefSeq	47,846

**Table 6:** Detailed Feature Lengths from NCBI Eukaryotic Pipeline.

Feature	Count	Mean length (bp)	Median length (bp)	Min length (bp)	Max length (bp)
Genes	28,166	11,149	4,361	56	677,970
All transcripts	59,938	3,654	2,773	56	106,526
mRNA	47,846	3,907	3,042	204	98,797
misc_RNA	2,018	3,844	2,824	138	22,974
tRNA	1,351	74	73	71	87
lncRNA	5,304	3,880	1,632	112	106,526
snoRNA	117	123	126	62	319
snRNA	378	142	141	56	196
rRNA	2,920	1,228	154	118	4,380
Single-exon	514	2,381	1,944	358	21,617
coding	514	2,381	1,944	358	21,617
CDSs	47,846	2,373	1,617	96	97,746
Exons	277,161	325	142	2	38,823
coding	260,368	299	140	2	38,823
non-coding	27,774	515	152	9	36,521
Introns	247,597	1,355	160	30	611,280
coding	235,861	1,207	152	30	611,280
non-coding	22,579	2,911	304	30	498,241

## Reuse potential

The new version of the *S. scovelli* genome opens doors to more accurate results by enhancing the comparative genome data analysis and facilitating the creation of robust tools for molecular genetic studies. We generated the original version of the genome to focus on the genetic mechanisms underlying the unique body plan among pipefishes and seahorses. This genome version takes us one step closer to uncovering these evolutionary mysteries and aids in answering other unknown features, such as the effects of sexual selection and mate choice systems on genome evolution.

## Data Availability

The genome is available on NCBI with the assembly accession number GCA_024217435.2. The genome is annotated via the NCBI eukaryotic genome annotation pipeline, and the annotation report release (100) is available here. Several smaller contigs and contaminant microbes were removed in the annotation pipeline yielding a more robust genome assembly. The sequence identifier for the chromosome-level scaffolds is available in the GigaDB [28]. The NCBI Bioproject accession number is PRJNA851781, the raw Hi-Fi sequence accession is SRR19820733, the Hi-C sequence accession is SRR22219025, and the RNA-Seq sequence files from various tissues are SRR20438584–SRR20438604. Additional data is available in the GigaDB [28].

## Declarations

### List of abbreviations

BUSCO: Benchmarking Universal Single-Copy Orthologs; CCS: Circular Consensus Sequence; Gb: Giga basepair; Hi-Fi: High-Fidelity; Mb: Mega basepair; NCBI: National Center for Biotechnology Information; not: nucleotide; QUAST: Quality Assessment Tool; QV: Quality Value; SMRT: Single Molecule Real Time; University of Oregon Genomics and Cell Characterization Core Facility (UOGC3F).
